# A low-complexity region in the YTH domain protein Mmi1 enhances RNA binding

**DOI:** 10.1074/jbc.RA118.002291

**Published:** 2018-04-25

**Authors:** James A. W. Stowell, Jane L. Wagstaff, Chris H. Hill, Minmin Yu, Stephen H. McLaughlin, Stefan M. V. Freund, Lori A. Passmore

**Affiliations:** From the MRC Laboratory of Molecular Biology, Cambridge CB2 0QH, United Kingdom

**Keywords:** RNA-binding protein, intrinsically disordered protein, mRNA decay, meiosis, protein–nucleic acid interaction, nuclear magnetic resonance (NMR), deadenylation, exosome specificity factor, YT521-B homology, YTH domain

## Abstract

Mmi1 is an essential RNA-binding protein in the fission yeast *Schizosaccharomyces pombe* that eliminates meiotic transcripts during normal vegetative growth. Mmi1 contains a YTH domain that binds specific RNA sequences, targeting mRNAs for degradation. The YTH domain of Mmi1 uses a noncanonical RNA-binding surface that includes contacts outside the conserved fold. Here, we report that an N-terminal extension that is proximal to the YTH domain enhances RNA binding. Using X-ray crystallography, NMR, and biophysical methods, we show that this low-complexity region becomes more ordered upon RNA binding. This enhances the affinity of the interaction of the Mmi1 YTH domain with specific RNAs by reducing the dissociation rate of the Mmi1–RNA complex. We propose that the low-complexity region influences RNA binding indirectly by reducing dynamic motions of the RNA-binding groove and stabilizing a conformation of the YTH domain that binds to RNA with high affinity. Taken together, our work reveals how a low-complexity region proximal to a conserved folded domain can adopt an ordered structure to aid nucleic acid binding.

## Introduction

RNA-binding proteins regulate gene expression and can elicit profound phenotypic effects. In *Schizosaccharomyces pombe*, Mmi1 (meiotic mRNA interceptor 1) is essential for viability and represses the expression of transcripts required for entry into meiosis during normal vegetative growth (1). Many meiotic transcripts, such as the mRNAs encoding the transcription factor Mei4 and the meiotic cohesion subunit Rec8, are transcribed but rapidly eliminated in a manner dependent on *cis*-acting RNA elements known as determinants of selective removal (DSRs)^3^ (1).

Upon entry into meiosis, the Mmi1 protein is repressed by Mei2 and the long noncoding *meiRNA. meiRNA* contains DSR elements that are thought to act as a molecular sponge to sequester Mmi1 from its targets (1–3). This stabilizes meiotic transcripts, restores their expression, and results in large changes in transcriptional and post-transcriptional regulation of gene expression (4, 5).

Mmi1 contributes to multiple mechanisms of repression to ensure strict control over its targets. First, it induces the formation of heterochromatin islands on some repressed meiotic genes (6, 7). Second, Mmi1 targets meiotic transcripts for degradation by the nuclear exosome (1, 8, 9). Finally, Mmi1 binds the Ccr4–Not complex, which promotes deadenylation of DSR-containing target RNAs *in vitro* (10, 11). The relationship between these mechanisms of repression is unclear. Mmi1-mediated repression also extends to nonmeiotic transcripts (12).

A C-terminal YTH RNA-binding domain in Mmi1 recognizes U*N*AAAC motifs (where *N* is any ribonucleotide) that are present in multiple copies within DSRs (1, 3, 12). YTH domains are found in a variety of eukaryotic proteins (13, 14) and, in some cases, they mediate specific interactions with *N*6-methyladenosine (m6A) (15–17). Crystal structures have revealed that m6A is recognized in a deep pocket of the YTH domain by a cage of aromatic residues (18–20). Surprisingly, Mmi1 binds DSR RNA using a divergent mechanism on a surface that is distinct from the m6A-binding pocket, including extended protein sequences located upstream and downstream of the YTH domain (21, 22).

**Figure 1.**
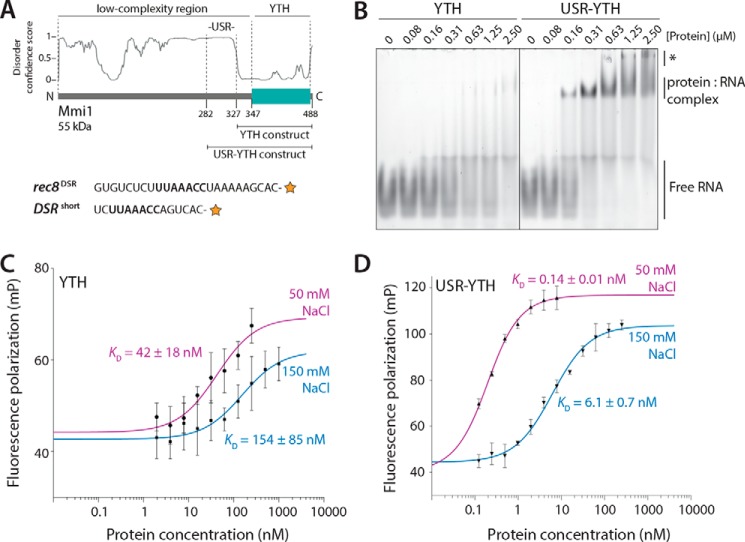
**The USR increases the affinity of interaction of the Mmi1 YTH domain with RNA.** *A, top,* schematic diagram of Mmi1 domain architecture, with the YTH and USR–YTH constructs indicated. The disorder prediction from DISOPRED3 is shown. *Bottom,* RNAs used for EMSAs (*rec8^DSR^*) and fluorescence polarization (*DSR^short^*). The *orange star* represents the 3′-fluorescein label, and the UNAAAC motif is in *bold. B,* USR–YTH construct binds RNA more stably than the YTH domain alone. A fluorescently-labeled *rec8^DSR^* RNA containing the Mmi1 DSR motif was analyzed by EMSA after incubation with purified proteins at the indicated concentrations. Binding was analyzed by native PAGE. Free RNA, shifted protein–RNA complex, and higher-order supershifted complexes (*asterisk*) are indicated. *C* and *D,* fluorescence polarization assays of YTH (*C*) and USR–YTH (*D*) binding to *DSR^short^* RNA (used at 0.1 nm). Calculated *K_D_* values are indicated, and *error bars* are the standard deviation of five biological replicates (each with three technical replicates).

Outside of its YTH domain, Mmi1 is primarily composed of a low-complexity amino acid sequence (residues 1–347) with no predicted secondary structure (Fig. 1*A*). This is consistent with a large proportion of RNA-binding proteins (up to one-third) containing substantial intrinsically disordered regions (IDRs), compared with the remainder of the proteome (23). IDRs can facilitate RNA binding or formation of higher order complexes (24, 25). We previously showed that the first 50 amino acids of Mmi1 contain a low-complexity region that is critical for binding Ccr4-Not (10). The remainder of the intervening polypeptide between this and the YTH domain has no attributed function.

Here, we show that a low-complexity region proximal to the Mmi1 YTH domain is required for stable RNA binding. Biophysical and structural analyses show that this region undergoes dynamic changes on RNA binding and decreases the off-rate for DSR RNA substantially. Thus, a low-complexity region proximal to a folded RNA-binding domain can strongly influence interactions with RNA substrates.

## Results

### A low-complexity region proximal to the Mmi1 YTH domain stabilizes the interaction with RNA

To investigate the interaction between the Mmi1 YTH domain and RNA, we performed electrophoretic mobility shift assays (EMSAs) with a fluorescently-labeled RNA substrate composed of a DSR sequence from the *rec8* 3′-UTR (*rec8^DSR^*, Fig. 1*A*). Full-length Mmi1 could only be expressed in small quantities and was difficult to purify to homogeneity. Thus, we tested binding with an Mmi1 YTH domain construct encompassing the RNA-binding domain, plus an additional 20 N-terminal amino acids with no predicted secondary structures that are nevertheless ordered in a published crystal structure and contact RNA (*residues 327–488,* Fig. 1*A*) (22). Although free RNA disappeared upon addition of increasing amounts of the YTH protein, a stable protein–RNA complex was not observed in the gel (Fig. 1*B*, *left panel*). It is likely that the YTH protein binds RNA but dissociates during electrophoresis, *i.e.* the off-rate is too fast to observe complex formation by EMSA.

To determine whether additional N-terminal sequence elements are involved in RNA binding and stabilization of the complex, we used a longer construct, including residues 282–488 (Fig. 1*A*). Residues 282–326 are largely hydrophilic and include 10 serines, a lysine/arginine-rich stretch (residues 301–309), and three prolines (residues 313–315). Purified Mmi1(282–488) behaved well in solution: nano-differential scanning fluorimetry (nano-DSF) and circular dichroism (CD) spectroscopy data were consistent with a folded YTH domain (Fig. S1). Furthermore, size-exclusion chromatography coupled to multiangle light scattering (SEC-MALS) showed that it was monomeric and monodisperse in solution (Fig. S2). When we analyzed RNA binding by EMSA using this construct, a stable RNA–protein complex was visualized as a better-defined band on the gel (Fig. 1*B*, *right panel*). Therefore, the upstream sequence proximal to the YTH domain likely increases the binding affinity of the YTH domain for DSR RNA. We termed this 45-amino acid segment the “upstream stabilization region” (USR).

To quantitatively measure the binding affinity (*K_D_*) of YTH and USR–YTH proteins for RNA, we used fluorescence polarization with a short 15-mer DSR-containing substrate (*DSR^short^*). Longer RNAs exhibit secondary binding events at higher concentrations, observed as a supershifted band in the EMSA (Fig. 1*B*, *asterisk*). We used increasing protein concentrations and buffer containing either 50 or 150 mm NaCl and measured the change in fluorescence polarization of the labeled RNA. The resulting equilibrium binding curves were fitted with a quadratic single-site binding curve. This revealed that at 150 mm NaCl, the binding affinity (*K_D_*) of YTH was 154 ± 85 nm (Fig. 1*C*). In comparison, an earlier study reported a binding affinity of 440 nm for Mmi1 residues 322–488, and 390 nm for residues 316–499, with a 10-mer RNA using isothermal titration calorimetry (22). In contrast, the USR–YTH construct had a binding affinity of 6.1 ± 0.7 nm, ∼25-fold stronger than YTH (Fig. 1*D*). A similar trend was observed at lower salt (Table 1). Therefore, the USR stabilizes the interaction between the YTH domain and RNA.

**Table 1:** Comparison of calculated binding parameters for Mmi1-RNA interactions

Construct (residues)	Fluorescence polarization, calculated *K_D_* (nm)		SwitchSENSE		
	(50 mm NaCl)	(150 mm NaCl)	Calculated *K_D_* (nm) (40 mm NaCI)	*k*_on_ (× 10^5^ m^−1^ s^−1^)	*k*_off_ (×10^−3^ s^−1^)
YTH (327–488)	42 ± 18	154 ± 85	16 ± 2	3.0 ± 0.4	4.90 ± 0.04
USR-YTH (282–488)	0.14 ± 0.01	6.1 ± 0.7	0.090 ± 0.006	8.3 ± 0.5	0.075 ± 0.0005
YTH-ΔC (327–483)			160 ± 80	12 ± 5	190 ± 20
YTH-ΔN (347–488)			15,000 ± 3,000	0.81 ± 0.13	1200 ± 200
YTH-ΔNΔC (347–483)			51,000 ± 34,000	0.38 ± 0.25	1900 ± 700

### The USR slows the off-rate for Mmi1 binding to RNA

**Figure 2.**
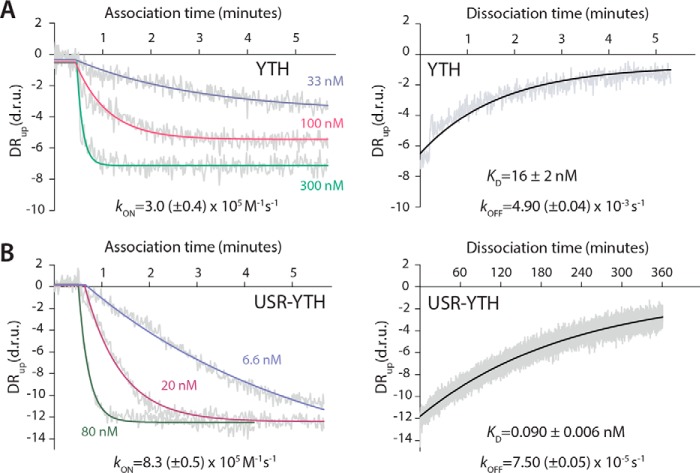
**The USR slows the off-rate of the YTH domain for RNA.** Association and dissociation kinetics were calculated for the YTH (*A*) and USR–YTH (*B*) constructs. SwitchSENSE association binding curves at the indicated protein concentrations, plotted as dynamic response (*DR_up_*) in dynamic response units (*d.r.u.*) *versus* time are shown on the *left*. Dissociation curves, generated by flowing buffer across the chip surface saturated with YTH or USR–YTH protein, are shown on the *righ*t. Dynamic response (*gray*) is the integrated fluorescence intensity between 2 and 6 μs. Fitted exponential decay curves are shown with calculated rate constants and standard errors.

We used SwitchSENSE technology to quantify the kinetics of the interaction between DSR-containing RNA and the Mmi1 constructs. SwitchSENSE measures the dynamics of protein binding to electro-switchable DNA nanolevers on a chip (26–28). Chimeric oligonucleotides that contained the 15-mer *DSR^short^* sequence followed by 42 deoxyribonucleotides complementary to the single-stranded DNA nanolever on the surface were used to functionalize the chip. Protein was then flowed across the surface, and the change in the switching dynamics of the nanolevers was measured. We fitted the resulting association and dissociation curves with single phase exponential decay curves (Fig. 2).

SwitchSENSE measurements revealed similar on-rate constants of ∼3.0 × 10^5^ m^−1^ s^−1^ for YTH and ∼8.3 × 10^5^ m^−1^ s^−1^ for USR–YTH. In contrast, the off-rate for YTH was 2 orders of magnitude faster than for USR–YTH: ∼3.5 × 10^−3^ and ∼7.5 × 10^−5^ s^−1^, respectively.

Dissociation constants calculated using these on- and off-rate constants agree with the equilibrium binding curves from fluorescence polarization at similar ionic strengths (Table 1). Thus, the USR increases the stability and longevity of the Mmi1 YTH domain–DSR RNA complex.

### The USR influences the chemical environment of the Mmi1 C terminus

**Figure 3.**
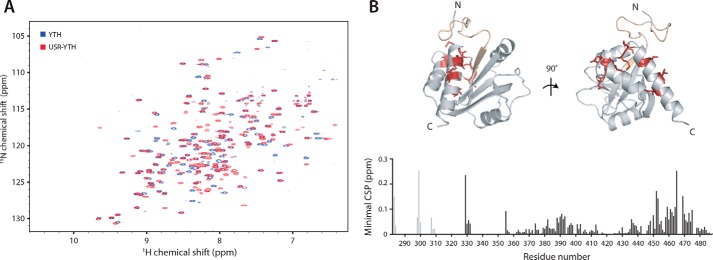
**The USR alters the chemical environment of the C terminus of Mmi1.** 2D NMR spectral analysis of YTH and USR–YTH constructs was performed. *A,* overlay of ^1^H-^15^N BEST-TROSY spectra of YTH and USR–YTH constructs. *B,* nearest neighbor chemical shift perturbation (*CSP*) maps of the assigned USR–YTH construct *versus* the unassigned YTH construct (*bottom*). *Lighter gray peaks* denote residues present in the USR–YTH construct but not the YTH construct. Regions that have large chemical shift differences (above 0.1 ppm; *red*) or are line broadened (*yellow*) are mapped onto the crystal structure of the Mmi1 YTH domain (*top*).

Because the USR has no predicted secondary structure, we used NMR spectroscopy to investigate its properties in solution. We recorded ^1^H-^15^N BEST-TROSY spectra, which correlate the backbone amide nitrogen and hydrogen resonance frequencies to give a spectral “fingerprint” of the protein, with one cross-peak possible for each amino acid in the protein except prolines. The spectrum of USR–YTH showed good dispersion, suggesting a folded construct with both α-helical and β-sheet secondary structure (Fig. 3*A*). Even though ∼50 residues have low sequence complexity, there was no evidence of substantial random coil regions, which would typically manifest as relatively sharp peaks at 8–8.5 ppm in the proton dimension. Thus, the USR may have some order.

We next recorded three-dimensional spectra for backbone assignment of USR–YTH. Spectra of ^13^C-^15^N–labeled protein were incomplete and had low signal intensities. This suggested either faster overall relaxation as a result of a large hydrodynamic radius or line broadening caused by conformational averaging. Overall relaxation behavior improved with deuteration of nonlabile carbon-attached protons; however, samples obtained from expression in deuterated media suffered from a lack of “back-exchange” for residues later identified to be from the β-sheet. These slowly-exchanging amide protons yielded signals only after incubation of the sample with a chaotropic agent (4 m urea) followed by dialysis to remove urea. Taken together, this indicates a highly dynamic domain with a rigid core consisting of the β-sheet and more flexible regions where only broad or no signals were observed. In total, only ∼60% of residues were assignable. This included only 14 confirmed residues in the low-complexity region, suggesting that the remainder of the signals from this region are missing due to line broadening; there were ∼50 fewer cross-peaks than expected, even in the BEST-TROSY experiment with nondeuterated protein. The lack of narrow, intense signals attributable to unstructured regions due to quasi-individual mobility of residues means the results obtained here suggest a sampling of interconverting conformations on a micro- to millisecond time scale.

We compared the assigned USR–YTH spectrum with an unassigned YTH spectrum using nearest neighbor minimal chemical shift perturbation maps (Fig. 3). These maps show the distance from a USR–YTH peak to the closest peak in the unassigned YTH spectrum (which may or may not represent the same residue) and thus indicate which amide groups have different chemical environments, as well as the smallest shift that could occur. Some of the largest chemical shift differences occur in residues in the C-terminal helix, consistent with the N-terminal USR being in close proximity to (or influencing the chemical environment of) residues in the C terminus of the protein. Together, our observations suggest that the USR likely exhibits a preferred conformation in solution in the absence of RNA.

### The USR and the YTH domain interact via a network of hydrophobic contacts

In a previous crystal structure of the Mmi1 YTH domain (residues 322–488), most of the USR was not included (22). DSR RNA was bound in a pseudo-helical conformation in a groove generated by the central β-sheet and the C-terminal α-helix using both positively-charged and hydrophobic surfaces. Residues N- and C-terminal to the annotated YTH domain contact the 3′ and 5′ ends of the UNAAAC motif, respectively. We hereafter refer to these regions of Mmi1 as the N- and C-clamps. This structure revealed the molecular basis for recognition of specific RNA sequences, but there was no structural information available on how the USR increases the affinity for RNA. The USR region could enhance RNA binding through additional direct interactions or by influencing the known RNA-binding interface. To distinguish these possibilities, we attempted to crystallize USR–YTH in complex with RNA.

**Figure 4.**
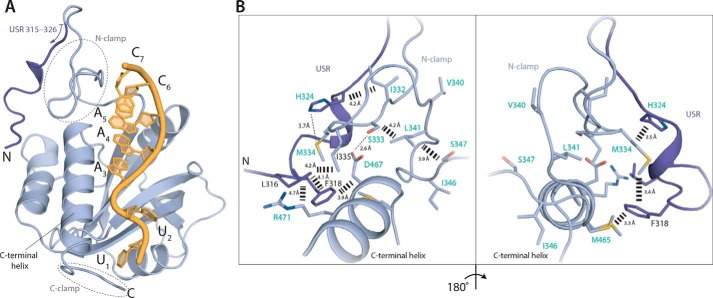
**A network of interactions is formed between the USR, N-clamp, and core YTH domain.** *A,* co-crystal structure of Mmi1 USR–YTH (residues 315–488 modeled) with DSR RNA. Cartoon representation shows protein in *blue* and RNA in *yellow*. The USR is shown in *darker blue. B,* conformation of the USR is stabilized by hydrophobic contacts with the core YTH domain and N-clamp. Residues labeled in *turquoise* either appear or show large chemical shift changes on RNA binding in NMR experiments (see Fig. 5).

We were not successful in crystallizing the USR–YTH construct itself, but removal of an additional 19 residues from the N terminus allowed us to crystallize Mmi1 residues 301–488 bound to a 7-mer DSR RNA (UUAAACC). We determined its X-ray crystal structure to 1.4 Å resolution (Table S1) and built an atomic model for residues 315–488 and all seven nucleotides (Fig. 4). We also determined a structure of apo-Mmi1 residues 327–488 (Table S1).

The core YTH domain of our structure of Mmi1-RNA is similar to apo-Mmi1 and to previously reported structures (21, 22) with a central five-stranded β-sheet surrounded by four α-helices. Part of the N-terminal USR region of the polypeptide, which was line broadened in NMR spectra (making structural analysis of this region impossible by NMR), was ordered in the crystal structure and folds back to contact the back of the YTH domain (Fig. 4*A*). This results in a network of hydrophobic interactions involving residues Leu-316, Phe-318, and His-324 of the USR, Met-334 and Ile-335 from the N-clamp, and Met-465 and Arg-471 from the YTH domain (Fig. 4*B*). Interestingly, the low-complexity N-clamp region adopted a similar conformation in two structures of Mmi1 determined in the absence of RNA (this work and Ref. 21). Both apo- and RNA-bound structures have crystal-packing contacts in their N-terminal regions (Fig. S3). This could stabilize the conformation of the low-complexity region, even in the absence of RNA. Furthermore, structural alignments of all known Mmi1 YTH domain structures show that the core fold is highly similar (r.m.s.d. <0.5 Å), but the N- and C-clamps are more divergent (Fig. S4, *A* and *B*). This observation is also consistent with higher relative B-factors in these regions (Fig. S4*C*). In summary, our structure shows that part of the low-complexity USR of Mmi1 (residues 315–326) lacks secondary structure elements but adopts a defined fold on RNA binding, forming contacts with the YTH domain.

### Dynamic changes in Mmi1 USR–YTH on RNA binding

Because we were unable to crystallize the full-length USR–YTH construct and the USR may adopt more than one conformational state, we further studied RNA binding using NMR. Again, we performed backbone experiments on USR–YTH, this time bound to unlabeled 19-mer DSR-containing RNA. We could assign ∼70% of the residues in the RNA-bound form. In contrast to the unbound protein, a peak count of the ^15^N-^1^H spectrum showed that almost all residues were represented by a cross-peak.

**Figure 5.**
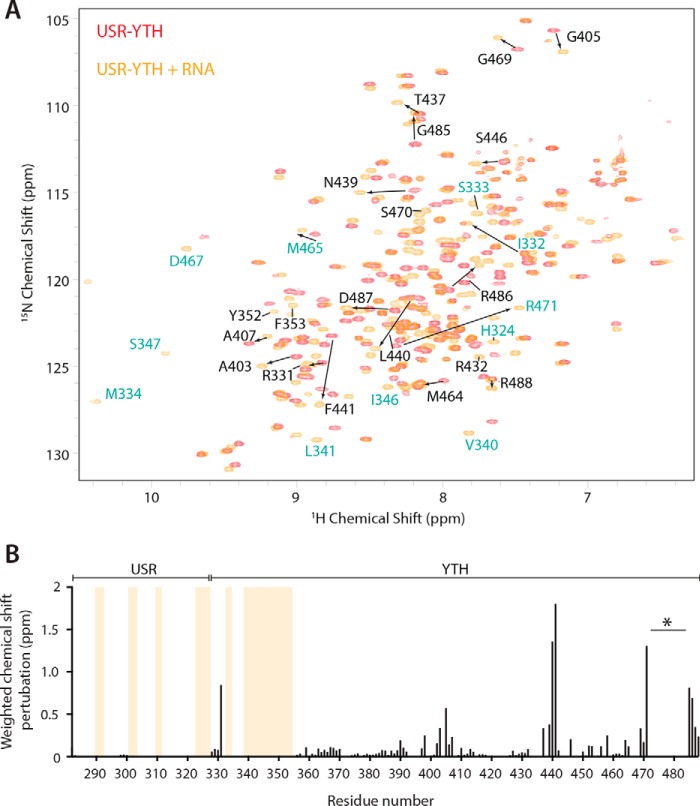
**The USR becomes more ordered on RNA binding.** *A,* overlay of the ^1^H-^15^N BEST-TROSY spectra of USR–YTH Mmi1 in the absence (*red*) and presence (*orange*) of RNA. Selected assigned peaks that show large chemical shift perturbations (denoted with *arrows*) or appear on RNA binding are labeled. Residues labeled in *turquoise* are shown in Fig. 4*B. B,* plot showing the chemical shift perturbations per residue. *Yellow bars* indicate peaks that are only present in the RNA-bound form. The gap in the C-terminal region (*asterisk*) is due to line broadening in the presence of RNA. Other missing signals in the core YTH domain fold are due to incomplete back-exchange of the deuterated protein.

Next, to map residues whose chemical environments change on RNA binding, we compared the assigned NMR amide group chemical shifts in the spectra of USR–YTH in the presence and absence of RNA. We observed some large-scale shifts and the appearance of new peaks on RNA binding (Fig. 5*A*). When these chemical shift perturbations are mapped onto the crystal structure, few changes are found on the RNA-binding central β-sheet, but instead they are located mainly in the N- and C-terminal regions (Fig. 5*B*). Surprisingly, 31 additional amide peaks of the low-complexity region could be assigned in the presence of RNA (Fig. 5*B, yellow lines*). Some of these correspond to residues not observed in the crystal structure. Thus, in the presence of RNA, it is likely that the conformation of the USR and N-clamp become even more constrained, reducing the line broadening in NMR spectra and thereby leading to the appearance of additional protein amide peaks.

RNA binding to USR–YTH also leads to loss of amide signals corresponding to the C-terminal helix of Mmi1 (again precluding NMR structural analysis) (Fig. 5*B*, *asterisk*), suggesting that, in the presence of RNA, this region may adopt multiple conformations or is contacted transiently by the USR. Examination of relative B-factors from our crystal structures suggests that the final C-terminal helix is relatively rigid when bound to RNA (Fig. S4*C*). The N terminus of the modeled density in our crystal structure is in a position such that residues further upstream could extend toward the C-clamp (Fig. 4*A*), consistent with transient interactions with the USR, resulting in the line broadening seen by NMR.

**Figure 6.**
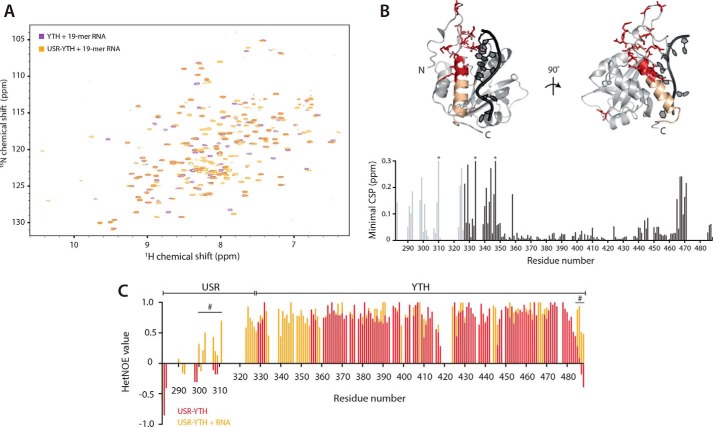
**The USR influences the RNA-bound N- and C-clamps.** 2D-NMR spectral analysis of YTH and USR–YTH constructs bound to a 19-mer DSR-containing RNA. *A,* overlay of ^1^H-^15^N BEST-TROSY spectra. *B,* nearest neighbor CSP maps (*bottom*). *Lighter gray peaks* denote residues present in the USR–YTH construct but not the YTH construct. *Asterisks* mark chemical shifts >0.3 ppm. Regions showing large chemical shift differences (above 0.1 ppm; *red*) or line broadening (*yellow*) are mapped onto the crystal structure of the Mmi1 YTH domain (*top*). *C,* plot of ^1^H-^15^N heteronuclear NOE values per residue in the absence (*red*) and presence (*orange*) of RNA. Positive values approaching 1 represent more ordered amide backbone regions. Peak absences represent residues that are not assigned. Many N-terminal residues that appear on RNA binding have hetNOE values that suggest they are ordered. The *hash symbol* marks some regions, assigned in both the absence and presence of RNA, with enhanced rigidity in the presence of RNA.

We also used minimal chemical shift perturbation maps to compare the assigned USR–YTH/RNA spectra to unassigned spectra of YTH bound to RNA to examine how the USR influences binding (Fig. 6, *A* and *B*). There are several changes in the USR–YTH spectrum, clustered at the N and C termini. For example, the chemical environments of Asp-467 and Arg-471 at the top of the C-terminal helix change. These residues contact the N-clamp and USR, respectively (Fig. 4*B*). In addition, a cluster of residues within the N-clamp, including Asn-344, Ile-346, and Ser-347, are in a different chemical environment, possibly because of increased flexibility of this region in the absence of the USR.

These data are consistent with the USR contributing to the interaction with RNA via stabilizing the conformation and reducing the flexibility of the N-clamp in solution. The transient nature of this interaction and the subsequent line broadening made formal determination of this conformation elusive by the traditional NMR structural technique of NOE analysis; however, to determine how backbone dynamics are altered, we performed ^1^H-^15^N heteronuclear NOE (hetNOE) experiments that are sensitive to motion on the picosecond time scale. When a residue is part of a rigid secondary structure element, such as a β-sheet, the hetNOE value is ∼0.8. Residues from more flexible parts of the protein have lower hetNOE values, often reaching negative values at highly flexible N and C termini. The hetNOE experiments show that the N- and C-terminal regions of USR–YTH become more ordered on RNA binding (Fig. 6*C*). Specifically, the N-clamp, the USR, and residues 485–488 are flexible or unassigned in the absence of RNA but have enhanced rigidity in the presence of RNA, confirming our indirect observation that the addition of RNA stabilizes the dynamic nature of the protein resulting in more residue peaks being present in the NMR data. In addition, the first β-strand of the core YTH domain is exchange-broadened in the absence but not in the presence of RNA, suggestive of enhanced rigidity upon binding. Together, our observations show that the USR influences the chemical environment of both the N- and C-clamps to enhance RNA binding.

### The USR adopts the same conformation with longer RNA substrates

The RNA used in NMR experiments (19 nucleotides) is longer than the RNA used in crystallographic experiments (7 nucleotides). To determine whether the conformation of the USR and N-clamp are influenced by RNA length, we compared spectra of the USR–YTH construct bound to 19- and 7-mer DSR-containing RNAs (Fig. S5, *A* and *B*). There are no major differences between the spectra or the minimal perturbation maps. Two clusters of residues close to the C-clamp region of the core YTH domain show moderate changes. Binding of the 5′-end of the longer RNA in the C-clamp may involve additional contacts, altering the chemical environment of this particular region. Alternatively, an interaction of the RNA with the USR may contribute to these changes.

To determine whether nucleotides outside the UNAAAC motif are recognized by Mmi1, we determined a crystal structure of USR–YTH (residues 301–488) bound to an 11-mer RNA containing a DSR (Fig. S5, *C* and *D*; Table S1). We could model residues 312–488. In this structure, the longer RNA does not form any additional contacts with the YTH domain, and RNA binding is confined to the DSR motif. Furthermore, the conformation of the N-terminal USR is largely similar to those seen in the other structures.

### Mmi1 residues 282–346 are critical for DSR binding

Together, binding assays, NMR, and X-ray crystallography suggested that residues 282–346 (which include the USR) contribute structurally to the interaction with RNA. In addition, the C-terminal residues of Mmi1 have a different chemical environment upon RNA binding (Fig. 5*B*) We used truncation mutants to examine the effects of these regions on RNA binding *in vitro*. All truncation mutants could be purified after overexpression in *Escherichia coli* and were similar to the USR–YTH protein in CD and nano-DSF experiments (Fig. S1).

**Figure 7.**
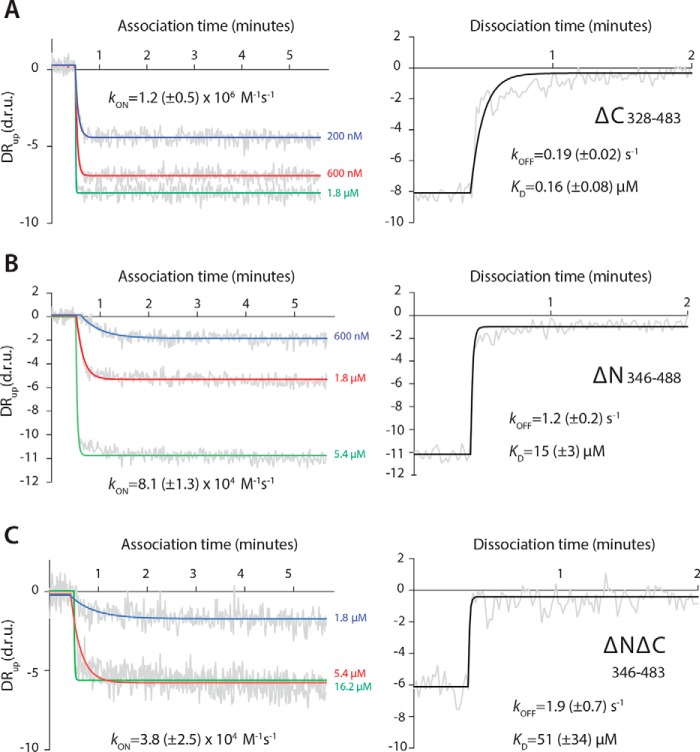
**Low-complexity regions of Mmi1 are critical for high-affinity binding to DSR RNA.** Binding kinetics for YTH-ΔC (*A*), YTH-ΔN (*B*), and YTH-ΔNΔC (*C*) constructs were determined using SwitchSENSE. Association (*left panel*) and dissociation (*right panel*) binding curves are shown, as dynamic response (*DR_up_*) *versus* time. Dynamic response is the integrated fluorescence intensity between 2 and 6 μs. Raw data are in *gray,* and fitted curves are in color or *black*.

A C-terminal truncation of the YTH construct (YTH-ΔC, residues 327–483) results in an ∼10-fold reduction in RNA-binding affinity compared with YTH (residues 327–488) (Fig. 7*A* and Table 1). Removal of the N terminus from YTH (YTH-ΔN, residues 347–488) reduces binding by 2 orders of magnitude (Fig. 7*B*). When both the N and C termini are deleted (YTH-ΔNΔC, residues 347–483), RNA binding is further abrogated (Fig. 7*C*). The RNA binding affinity of YTH-ΔNΔC, corresponding to the conserved YTH domain fold, is relatively weak (*K_D_* = 51 μm), underlining the key contribution of the low-complexity regions to RNA binding.

## Discussion

**Figure 8.**
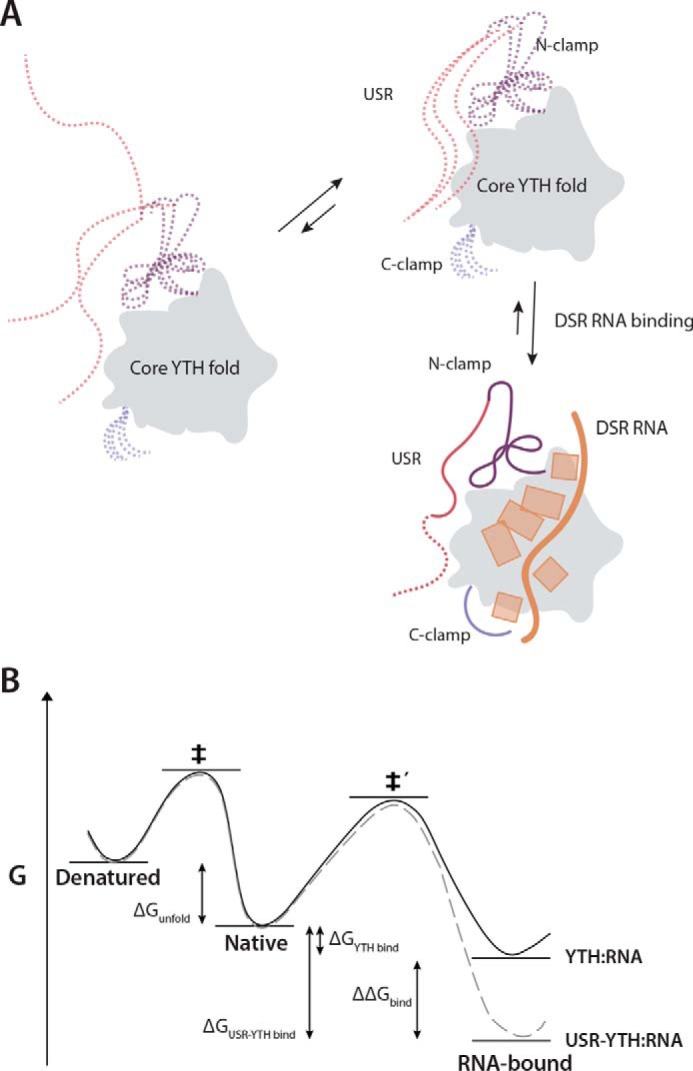
**Model for RNA binding by Mmi1.** *A,* schematic diagram showing the contribution of different regions of Mmi1 to DSR RNA binding. The core, conserved YTH fold acts as a platform for RNA binding but is unable to bind RNA with high affinity alone. The USR, N-clamp, and C-clamps have no secondary structure and are dynamic in the absence of RNA (*dashed lines*). The USR and N-clamp likely exist in an equilibrium between multiple conformers, at least one of which has high affinity for RNA. On RNA binding, these regions become more ordered and contact or reinforce contacts with RNA. RNA backbone and bases are shown in *orange. B,* thermodynamic model of USR–YTH interaction with RNA. The presence of the USR (*gray dashed line*) does not affect the thermodynamic stability of the native protein compared with YTH alone (*black line*). Hence, as there is no change in the energy for unfolding (Δ*G*_unfold_) to the denatured state, the apparent melting temperatures are the same. The USR lowers the energy of the RNA-bound complex (ΔΔ*G*_bind_) via changes in conformational dynamics and possibly a transient interaction with the RNA. Consequently, although the energy barrier for binding (native to ‡′) will be unaffected and similar association kinetics are observed with and without USR, the barrier for dissociation has increased, leading to significantly slower dissociation rates for USR–YTH/RNA compared with YTH/RNA.

Specific and high-affinity RNA binding are likely important for Mmi1 to efficiently repress expression of its target RNAs and prevent premature entry into meiosis. Here, we show that stable RNA binding by Mmi1 is dependent not only on its YTH domain but also on an upstream low-complexity region (residues 282–346). This N-terminal, low-complexity region does not adopt any formal secondary structure but forms a stably-folded platform encompassing the N-clamp and extending across the YTH domain to contact the C-terminal helix (Fig. 8*A*). Although this low-complexity region contributes only two residues to RNA-binding (residues Asn-336 and Arg-338), our truncation experiments demonstrate that it is critical for the interaction with DSR RNA. Residues 282–346 most likely further stabilize a helical conformation in the bound RNA, and the resultant base-stacking may thermodynamically drive stable binding.

Within the low-complexity region, the USR (residues 282–326) likely contributes to RNA binding indirectly. We were able to model 15 residues of the USR using crystal structures. The USR adopts a preferred conformation in solution: a network of interactions secures it onto the back of the core YTH domain (Fig. 4). The remaining part of the USR is likely more dynamic, but we investigated its properties using NMR. On RNA binding, the chemical environment of the USR changes (Fig. 5), and the USR becomes even less dynamic (Fig. 6*C*), thus stabilizing its conformation (Fig. 8). Substantial portions of the USR correspond to broadened or missing signals, suggesting that this region is not in a random-coil conformation but samples a number of structures on the micro- to milli-second time scale. Taken with the flexibility of those observable residues in the USR without RNA by hetNOE analysis, this slow inter-conversion of states is not likely to include a substantial amount of formal secondary structure. Light-scattering experiments also support this conclusion, with inclusion of the USR increasing the hydrodynamic radius from 2.2 to 2.8 nm (Fig. S2). Interestingly the radius decreases to 2.6 nm in the presence of RNA suggesting a compaction of the low-complexity region. Finally, CD spectra recorded for all the constructs showed a similar line shape, although the magnitude of the signal at ∼225 nm increases as more of the low-complexity region is included (Fig. S1*B*). This can be characteristic of proteins with a molten-globule conformation (29).

The USR most likely enhances RNA binding by reducing the dynamic motion of the YTH RNA-binding groove. The USR interacts with residues of both the N- and C-clamps of Mmi1, thus stabilizing the conformation of residues within these regions that contact RNA. This is supported by comparative NMR analysis of USR–YTH and YTH, showing that the chemical environments differ for both N-terminal low-complexity residues and residues in the C-terminal helix. The interaction between Mmi1 and the 3′-end of the DSR RNA is stabilized by the hydrophobic interface between the low-complexity region and the N terminus of the core YTH fold (Fig. 4).

In the presence of RNA, signals assigned to the C-terminal helix of the YTH domain become line broadened (Fig. 5*B*), precluding any comparison of the dynamics of this region when bound to RNA. Given the close proximity of the USR to the C terminus on RNA binding, this may be due to the formation of a dynamic interface with RNA in this region. In agreement with this, the N-terminal (residues 322–338) and C-terminal (residues 483–488) regions were found to be disordered in a previous crystal structure (22).

Residues 301–311 of the USR are not visible in our crystal structure, and signals from most of this region are line broadened in NMR experiments. This includes a basic region (Arg-301–Arg-309). Regions rich in arginine and lysine can contribute to RNA binding directly. For example, serine/arginine-rich regions are intrinsically disordered but are important for functional RNA binding (30, 31). Arginine and glycine repeats (RGG boxes) within a low-complexity region also contribute to RNA interaction, for example in the nuclear RNA export factor 1 (NXF1) (32, 33) and fragile X mental retardation protein (FMRP) (34, 35). Many DNA-binding proteins, including transcription factors, also contain IDRs that directly interact with DNA (36). Because of the lack of defined structures for IDRs, there are only a few examples of studies that reveal molecular details of their interaction with DNA/RNA. A solution structure of an RGG peptide from FMRP bound to G-quadruplex RNA reveals that basic residues insert into the major groove of the RNA duplex and form hydrogen bonds with bases (37). In another example, a crystal structure of thymine DNA glycosylase with an extended N terminus demonstrates that, although the structure of the N-terminal region is dynamic, it contributes to high-affinity DNA binding, partly through an arginine contact to DNA backbone phosphates (38, 39). In Mmi1, if the basic region transiently contacts RNA, it could be through either of these mechanisms: backbone phosphate contacts or interaction with bases. Three prolines (Pro-313–Pro-315) may constrain the basic region such that it is more likely to interact with RNA.

In addition to potential direct charge-mediated interactions with RNA, low-complexity regions can contribute to RNA binding indirectly by stabilizing a particular conformation of a folded domain, as we show here for Mmi1. RNA-binding proteins often contain IDRs proximal to a folded RNA-binding domain. Our model for RNA binding by Mmi1 is likely applicable to many of these other proteins. The canonical RNA-binding domains would provide specificity, whereas the IDRs contribute to RNA binding in a sequence-independent manner. The sequence of the USR is not conserved outside *Schizosaccharomyces* Mmi1 orthologues (Fig. S6 and S7), although a proximal intrinsically disordered region appears to be present in all YTH domain-containing protein clades examined (Fig. S8). IDRs are not constrained by 3D structure, and therefore their sequence may evolve more rapidly than the sequence of structured domains (40). It is possible that the IDRs in other RNA-binding proteins act similarly to increase their affinity for RNA, but sequence analysis in the absence of structural information makes comparative studies difficult.

More broadly, this function of a low-complexity region represents a general mechanism to stabilize a specific conformation of a globular protein. On binding to an interacting partner, IDRs can modulate conformational entropy and result in allosteric coupling (36, 41, 42). An ensemble of conformational states of a structured domain can be influenced by binding of an IDR, selecting for a certain conformation (43). In the case of Mmi1, the USR stabilizes a conformation of the YTH domain that binds RNA with high affinity. Such a mechanism has also been demonstrated in other proteins (42). For example, an IDR in the glucocorticoid receptor induces allosteric changes in the DNA binding domain to enhance DNA binding, likely by stabilizing a high-affinity conformation (44). Thus, Mmi1 is an example of a protein with a low-complexity region that can act as an enhancer element to fine-tune protein conformation.

## Experimental procedures

### Mmi1 YTH domain expression and purification

Genes were cloned into a modified pET28 vector and expressed in BL21Star cells as N-terminally tagged 3C protease-cleavable hexahistidine fusion proteins. Proteins were expressed for ∼18 h overnight at 18 °C. Cells were resuspended in lysis buffer (50 mm PIPES, pH 7.0, 1.0 m NaCl, 5% (w/v) glycerol, 1 mm TCEP, protease inhibitor mixture (Roche Applied Science), and 0.4 mm phenylmethylsulfonyl fluoride), lysed by sonication, and cleared using ultracentrifugation. Proteins were purified from lysate using batch binding with nickel-nitrilotriacetic acid–agarose, washed with lysis buffer, and eluted in low pH buffer (20 mm BisTris, pH 6.0, 300 mm NaCl, 250 mm imidazole, 5% (w/v) glycerol, and 1 mm TCEP). The His tag was removed by overnight cleavage at 4 °C with 3C protease. Cleaved protein was diluted 2-fold in 20 mm BisTris, pH 6.0, and 0.5 mm TCEP buffer before loading on a 5-ml HiTrap S (GE Healthcare) cation-exchange column. Protein was eluted using a gradient of 0.15–1.0 m NaCl over 12 column volumes. Fractions containing Mmi1 were loaded onto a Superdex 75 26/60pg size-exclusion column equilibrated with 20 mm PIPES, pH 7.0, 150 mm NaCl, and 0.5 mm TCEP. Purified Mmi1 was concentrated using a 10,000 MWCO Amicon Ultra-15 concentrator (Millipore) to ∼5 mg/ml for the short constructs or >20 mg/ml for the longer constructs.

### CD spectroscopy

Spectra were recorded on a Jasco J-815 CD spectrometer with a CDF-426S/15 temperature controller at 25 °C. Protein was diluted to 0.2 mg/ml in buffer (10 mm TAPS, pH 8.2, 40 mm NaCl, 0.5 mm MgCl_2_, 0.1 mm TCEP, 0.01% Brij-35) for assays. Spectra were recorded over 260 to 190 nm with a data pitch of 0.5 nm and scanning speed of 50 nm/min. Spectra plotted in Fig. S1B are the average of 20 accumulated scans.

### Differential scanning fluorimetry

Melt curves utilizing the intrinsic tryptophan and tyrosine fluorescence of the protein sample were recorded using a Prometheus NT.48 nano-DSF instrument (NanoTemper technologies). Protein was diluted to 0.2 mg/ml in binding buffer (as above) and recorded in nano-DSF grade standard capillaries at 50% fluorescence intensity between 15 and 95 °C with a temperature ramp rate of 2 °C/min.

### SEC-MALS

Experiments were performed using an Agilent 1200 series LC system with an on-line Dawn Helios ii system (Wyatt) with a QELS+ module (Wyatt) and an Optilab rEX differential refractive index detector (Wyatt). Detector 12 in the static light-scattering cell had been replaced with the QELS module. 100 μl of purified sample at 10 mg/ml was autoinjected onto a Superdex 75 increase 10/300gl column (GE Healthcare) and run at 0.5 ml/min. The molecular weights and hydrodynamic radii were calculated in ASTRA software (Wyatt).

### EMSAs

EMSAs were performed with fluorescently-labeled RNA oligonucleotides with increasing concentrations of Mmi1 YTH domain constructs. 2-Fold dilution series of proteins were prepared at 5× final concentration in protein dilution buffer (20 mm PIPES, pH 7.0, 150 mm NaCl, 0.5 mm TCEP). 10-μl binding reactions were assembled with the following components: 2 μl of protein, 1 μl of 500 nm RNA probe, 1 μl of 10× buffer (200 mm HEPES, pH 7.5, 1.0 m NaCl, 10 mm MgAc, 1.0 mm TCEP, 50% (w/v) glycerol), and 6 μl of diethyl pyrocarbonate H_2_O. After incubation for 15 min, samples were run on 10% TBE-PAGE. Gels were run at 100 V (∼15 mA starting current) for 90 min and visualized using a Typhoon FLA 7000.

### Fluorescence polarization

Fluorescence polarization was performed using fluorescently-labeled RNA oligonucleotides. 2-Fold protein dilution series were prepared at 10× concentration in dilution buffer (20 mm PIPES, pH 7, 100 mm NaCl, 0.5 mm TCEP). Protein was incubated at room temperature for 30 min with 0.1 nm 3′,6-carboxyfluorescein–labeled DSR-containing RNA (synthesized by IDT) in buffer containing 10 mm TAPS, pH 8.2 (+2 mm PIPES carried over from protein buffer), 40 or 140 mm NaCl (+10 mm NaCl carried over from protein buffer), 0.5 mm MgAc, 0.1 mm TCEP, and 0.01% Brij-35 in a total of 100 μl using 384-well low-flange black flat bottom nonbinding surface microplates (Corning). Fluorescence polarization was measured using a PHERAstar Plus (BMG Labtech). Dissociation constants were estimated using a quadratic function Prism 6.0 (GraphPad software). Error bars indicate the standard deviation of five biological replicates (each with three technical replicates).

### SwitchSENSE

Kinetic measurements were performed using a DRX series instrument (Dynamic Biosensors) with a MPC-48-2-Y1 chip (Dynamic Biosensors). Protein stock at the highest concentration to flow onto the chip was made in binding buffer (10 mm TAPS, pH 8.2, 40 mm NaCl, 0.5 mm MgAc, 0.1 mm TCEP, and 0.01% (w/v) Brij-35.) Automatic serial dilution to concentrations indicated in figures were made using the DRX instrument. Dynamic response of the chip was tested to determine the best measurement spots. Kinetic assays were designed in switchBUILD (Dynamic Biosensors) before being customized in switchCONTROL (Dynamic Biosensors). Binding experiments were performed at 20 °C at a flow rate of 30 μl/min with 5-min total association time measured using a single electrode. Dissociation kinetics were measured using binding buffer flowed over the chip surface for 10, 15, or 420 min (depending on the affinity). Data curves were fitted with single-phase exponential decay curves in Prism 6.0 (GraphPad Software), with associated standard errors. On-rates were calculated from three different concentrations. Dissociation constants were calculated using the observed on- and off-rates.

### NMR spectroscopy

Isotopically-labeled proteins were overexpressed in a modified K-MOPS minimal medium (45) containing ^15^NH_4_Cl. For 3D experiments, media also contained [^13^C]glucose. Medium was prepared in D_2_O for perdeuterated sample production so that nonlabile, carbon-attached protons are substituted with deuterons. All samples were dialyzed overnight against NMR buffer (20 mm PIPES, pH 6.8, 150 mm NaCl, 0.5 mm TCEP) before data collection. To improve back-exchange of deuterated protein, samples were incubated overnight at 20 °C in buffer containing 4 m urea, 20 mm PIPES, pH 6.8, 150 mm NaCl, 0.5 mm TCEP before undergoing two rounds of dialysis to remove the urea. For protein–RNA complexes, samples were mixed at a 1:1.2 protein/RNA molar ratio before dialysis.

All NMR data were collected at 298 K using either Bruker Avance II+ 700 MHz or Bruker Avance III 600 MHz spectrometers fitted with ^1^H{^13^C,^15^N} triple-resonance cryoprobes with 10% D_2_O added to each sample as a lock solvent. ^1^H-^15^N band-selective excitation short-transients transverse relaxation-optimized spectroscopy (BEST-TROSY) experiments were collected with an in-house optimized pulse sequence (46, 47). Preliminary data collection and analysis with a ^15^N,^13^C-labeled protein resulted in line-broadened peaks with poor signal intensity in the triple resonance experiments due to the dynamic properties of the USR–YTH construct. To improve data collection, triple-labeled, deuterated samples were used for residue assignment.

Assignment of backbone amide peaks was completed using the following standard TROSY triple resonance spectra: trHNCO and trHN(CA)CO experiments collected with 1024*64*128 complex points in the ^1^H, ^15^N, and ^13^C dimensions; trHNCA and trHN(CO)CA with 1024*64*128 complex points in the ^1^H, ^15^N, and ^13^C dimensions; and trHNCAB and trHN(CO)CACB with 1024*64*110 complex points in the ^1^H, ^15^N, and ^13^C dimensions. Backbone assignment was additionally aided by collection of nitrogen correlating trHN[CAN]NH and trHN[COCA]NNH spectra with 1024*80*80 complex points. Each backbone dataset was collected with nonuniform sampling set at 30–50% and processed using compressed sensing (48). All spectra were processed using the program Topspin 3.1 and analyzed using the program Sparky 3.115, with assignment aided by the program Mars (49). Weighted chemical shift perturbations were calculated using the equation √(Δδ^1^H)^2^ + (0.2(Δδ^15^N)^2^). ^15^N{^1^H}-hetNOE measurements were carried out using standard Bruker pulse programs, with interleaved on- or off-resonance saturation. The hetNOE values were calculated from peak intensities by taking the ratio *I*_on_/*I*_off_.

### X-ray crystallography

Crystals of a Mmi1 YTH domain construct (residues 327–488) concentrated to 5 mg/ml were grown using sitting drop vapor diffusion with a reservoir solution containing 20% (w/v) PEG 3350, 0.1 m BisTris, pH 5.5, and 0.2 m MgCl_2_. Crystals were cryo-cooled in mother liquor supplemented with 20% (w/v) glycerol before data collection at 100K at DLS beamline I03. Data were processed using the XIA2 pipeline (50) implementing DIALS for indexing and integration (51) and AIMLESS for scaling and merging (52). The structure was solved by molecular replacement with Phaser (53) using an NMR solution structure of YTHDF2 that had been truncated at both termini as the search model (PDB code 2YU6). Initial automated model building was carried out using phenix.autobuild (54) and Buccaneer (55). This was followed by iterative rounds of manual model building and refinement using COOT (56) and phenix.refine (57).

For co-crystallization, a YTH domain-containing construct composed of residues 301–488 was mixed with a 7-nt DSR RNA (UUAAACC) or an 11-nt DSR RNA (CUUUAAACCUA) at a 1:1.2 protein/RNA ratio. Screens were set up at ∼15 mg/ml, and crystals were grown in sitting-drop vapor-diffusion plates in 1:1 drops with reservoir containing 20% EtOH, 10 mm MgSO_4_, and 0.1 m Tris, pH 8.0, for the 7-nt RNA or 15% (w/v) PEG 8K and 0.1 m CHES, pH 9.5, for the 11-nt RNA. Diffraction data were recorded at ESRF beamline ID30-A or DLS beamline I02 for the 7-nt RNA and 11-nt RNA crystals respectively. Data were processed using GrenADES implementing XDS (58) or XIA2 pipeline (50) implementing DIALS (51), for indexing and integration, and AIMLESS (59) for scaling and merging. The structures were solved by molecular replacement using the apo-structure with Phaser (53), and model building and refinement was performed iteratively using COOT (56) and phenix.refine (57). RNA was modeled in COOT using RCrane (60, 61) after initial rounds of refinement.

Visualizations of structures were made using PyMOL. For structural alignments, all deposited Mmi1 structures were aligned against chain A of the YTH domain bound to 11-nt RNA using UCSF Chimera (62). This chain was used as a reference because it had the most modeled density of any of the structures. Structures that contained multiple chains in the crystallographic asymmetric unit were split into separate PDB files before alignment.

### Data availability

Backbone assignments of the USR–YTH protein in the absence and presence of the 19-mer RNA have been deposited with BMRB accession codes 27398 and 27399, respectively. Crystal structures were deposited in the PDB with accession codes 6FPP, 6FPQ, and 6FPX.

## Author contributions

J. A. S., S. M. F., and L. A. P. conceptualization; J. A. S., J. L. W., C. H. H., M. Y., S. H. M., and S. M. F. formal analysis; J. A. S., J. L. W., and S. M. F. investigation; J. A. S., J. L. W., and L. A. P. visualization; J. A. S., J. L. W., C. H. H., M. Y., S. H. M., and S. M. F. methodology; J. A. S. and L. A. P. writing-original draft; J. A. S. and L. A. P. project administration; J. A. S., J. L. W., C. H. H., S. H. M., S. M. F., and L. A. P. writing-review and editing; S. M. F. and L. A. P. supervision; L. A. P. funding acquisition.

## Supplementary Material

Supporting Information

## Footnotes

3 The abbreviations used are:

DSR: determinant of selective removal
BEST–TROSY: band-selective excitation short transients-transverse relaxation-optimized spectroscopy
CSP: chemical shift perturbation
DSF: differential scanning fluorimetry
dru: dynamic response units
hetNOE: heteronuclear nuclear Overhauser effect
IDR: intrinsically disordered region
m6A: *N*6-methyladenosine
QELS: quasi-elastic light scattering
SEC-MALS: size-exclusion chromatography coupled to multiangle light scattering
RGG: arginine-glycine-glycine
USR: upstream stabilization region
TCEP: tris(2-carboxyethyl)phosphine
BisTris: 2-[bis(2-hydroxyethyl)amino]-2-(hydroxymethyl)propane-1,3-diol
TAPS: 3-{[2-hydroxy-1,1-bis(hydroxymethyl)ethyl]amino}-1-propanesulfonic acid
CHES: 2-(cyclohexylamino)ethanesulfonic acid
nt: nucleotide
PDB: Protein Data Bank
FMRP: fragile X mental retardation protein.
