# Supplementary material for: A low-complexity region in the YTH domain protein Mmi1 enhances RNA binding
Source: J Biol Chem. 2018 Apr 25;293(24):9210–22. doi: 10.1074/jbc.RA118.002291 (PMC6005420; doi:10.1074/jbc.RA118.002291)
Supplement: Supporting Information [file supp_293_24_9210__index.html]

A low-complexity region in the YTH domain protein Mmi1 enhances RNA binding — Mmi1 low-complexity regions enhance RNA binding — A low-complexity region in the YTH domain protein Mmi1 enhances RNA binding — Mmi1 low-complexity region enhances RNA binding — Supporting Information 

# A low-complexity region in the YTH domain protein Mmi1 enhances RNA binding

## Supporting Information

- Supplementary information - 8 supplementary figures and 1 supplementary table
