# Supplementary material for: A low-complexity region in the YTH domain protein Mmi1 enhances RNA binding
Source: J Biol Chem. 2018 Apr 25;293(24):9210–22. doi: 10.1074/jbc.RA118.002291 (PMC6005420; doi:10.1074/jbc.RA118.002291)
Supplement: Supporting Information [file supp_RA118.002291_135756_2_supp_125716_p7njv6.pdf]

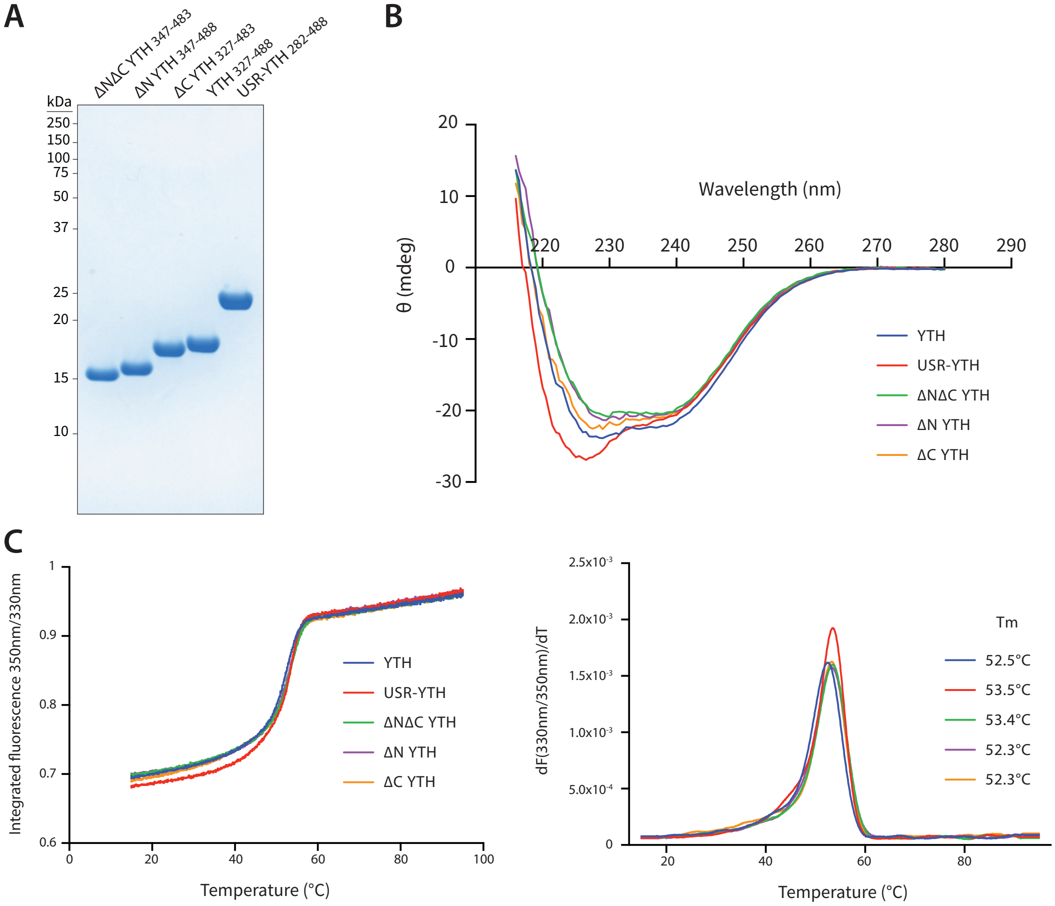

Figure S1. Characterization of Mmi1 constructs. (A) Coomassie-stained SDS-PAGE of purified constructs. Schematic diagrams of YTH and USR-YTH constructs are shown in Fig. 1A. ΔN corresponds to residues 347–488, ΔC is 327–483 and ΔNΔC is 347–483. (B) Circular dichroism spectra for the constructs shown in panel (A). (C) Nano differential scanning fluorimetry using intrinsic fluorescence. Left: The ratio of tryptophan fluorescence at 350 nm and 330 nm as a function of temperature. Right: The first derivative of the same data. The peaks are used to derive the melting temperatures (T_m) shown.

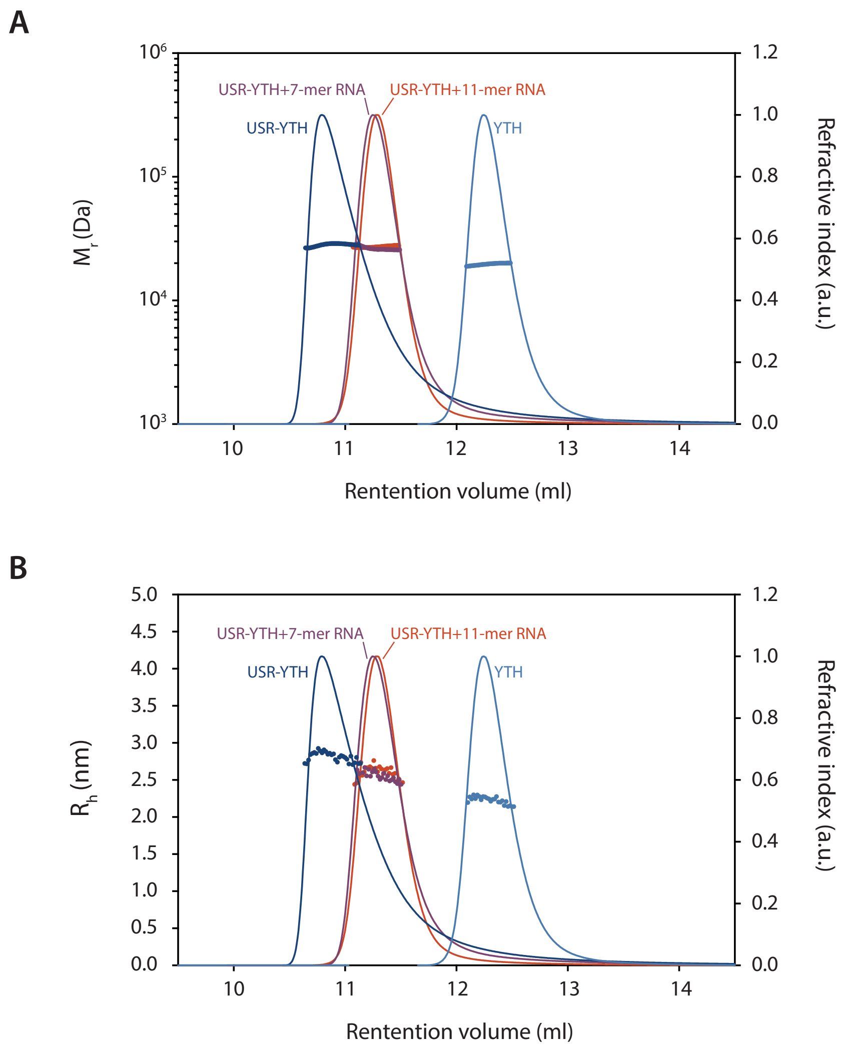

Figure S2. SEC-MALS with QELS analysis of YTH and USR-YTH constructs. Size exclusion coupled to multi angle light scattering (SEC-MALS) using quasi elastic light scattering (QELS) shows that the Mmi1 constructs used are monomeric and monodisperse in solution. (A) Calculated molecular weights (from MALS) for indicated samples with elution peaks represented by normalized refractive index as a function of retention volume on an in-line Superdex 75 increase 10/300gl gel filtration column. Calculated molecular weights (M_r) in kDa were as follows: YTH: 19.6 (theoretical 18.6), USR-YTH: 28.3 (theoretical 23.5), USR-YTH+7-mer RNA: 26.2 (theoretical 25.6), USR-YTH+11-mer RNA: 27.2 (theoretical 26.9). (B) Hydrodynamic radii (R_h) calculated using in-line dynamic light scattering (QELS module). The R_h value calculated for YTH (2.2 nm) is close to the theoretical R_h of a protein with the same number of residues (1). USR-YTH has a larger R_h (2.8 nm) consistent with a more elongated non-structured region. USR-YTH-RNA complexes have a slightly reduced R_h (2.6 nm) consistent with longer retention time on the gel filtration column. This could be due to a compaction of the unstructured region on RNA-binding.

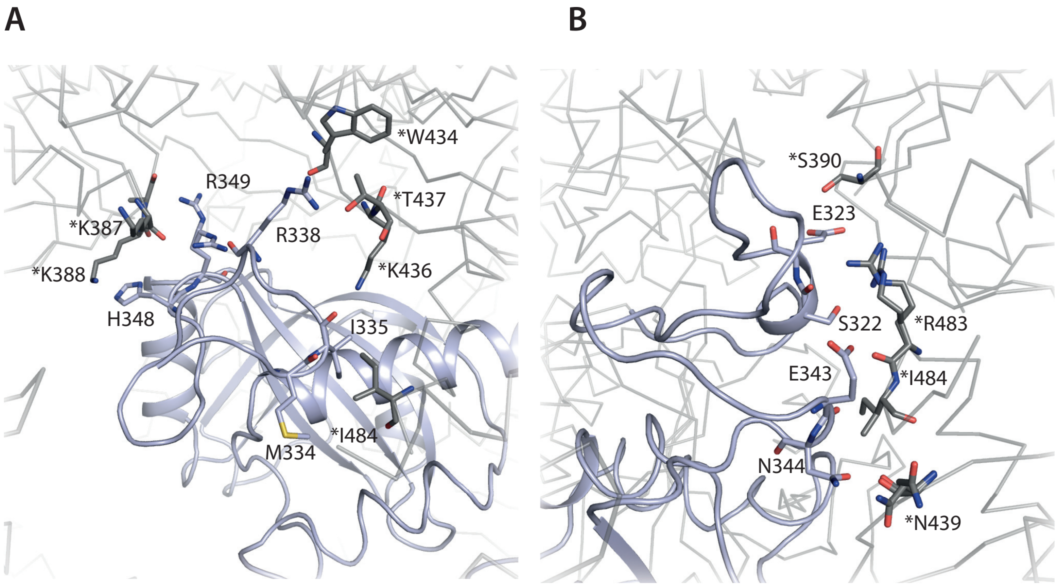

Figure S3. Crystal packing in Mmi1 crystal structures in the presence and absence of RNA could stabilize the conformation of the low-complexity region. (A) Cartoon representation highlighting residues involved in hydrogen bonding or hydrophobic interactions in the apo Mmi1 structure (A) or bound to 7-mer RNA (B). Residues marked with asterisks and colored in grey are from neighboring molecules.

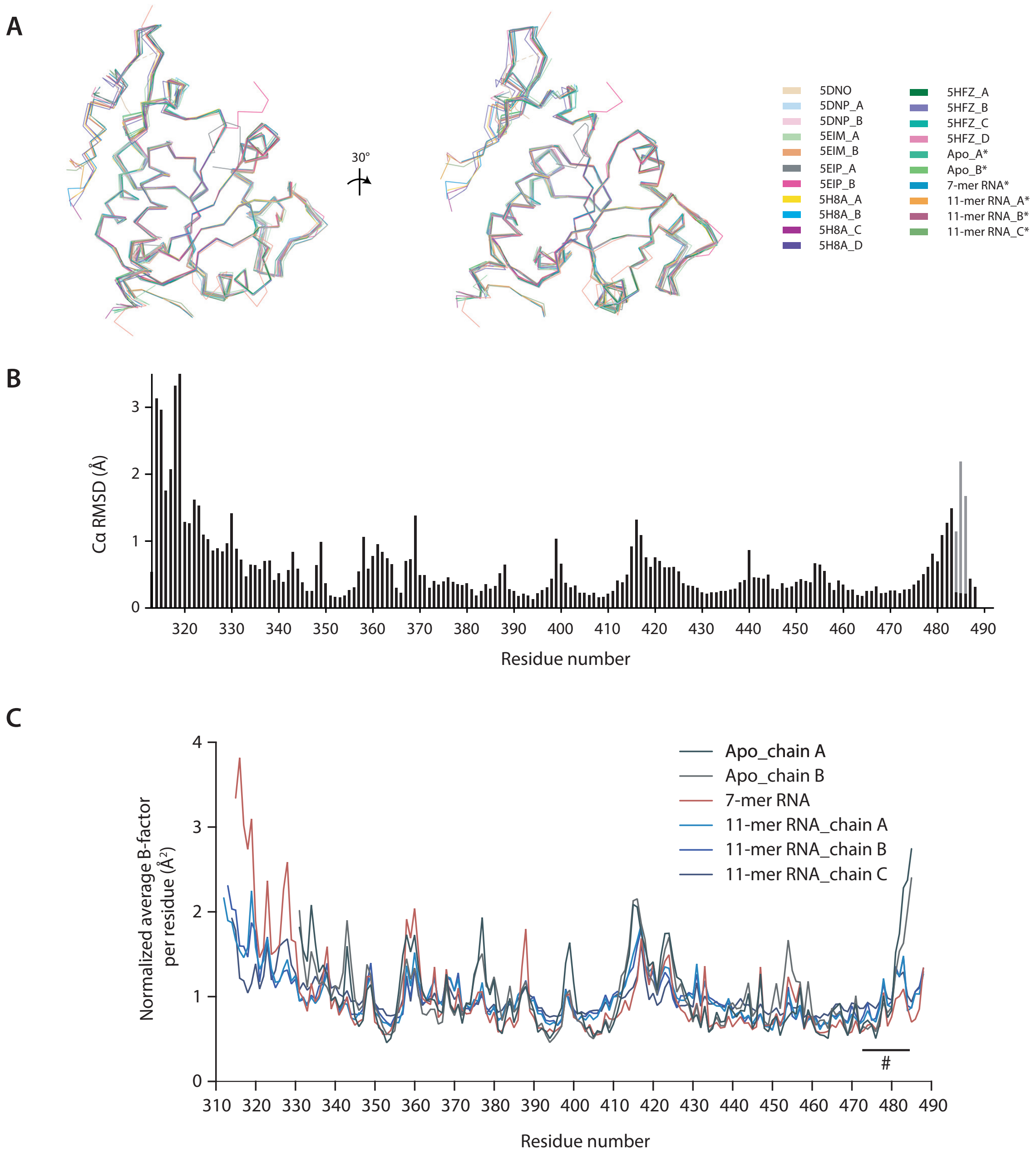

Figure S4. Alignment of Mmi1 YTH domain crystal structures. (A) PDB coordinates 5DNO, 5DNP, 5HFZ, 5EIM, 5EIP, 5H8A and the three structures from this study (marked with asterisks) were included in the analysis. Where multiple chains existed in the asymmetric unit, coordinates were split into separate files before alignment. All structures were aligned against the YTH domain structure co-crystallized with 11-mer DSR RNA since this contained the longest polypeptide chain. (B) Root mean squared deviations in Å per residue from alignment shown in panel (A). Grey lines at the C-terminus denote deviations in structures that are not RNA-bound. (C) Plot of average normalized B-factor per residue for structures in this study. Average B-factor for a given residue was normalized to the overall average B-factor. The C-terminal region that is line broadened in the presence of RNA in NMR studies is marked with a hash symbol.

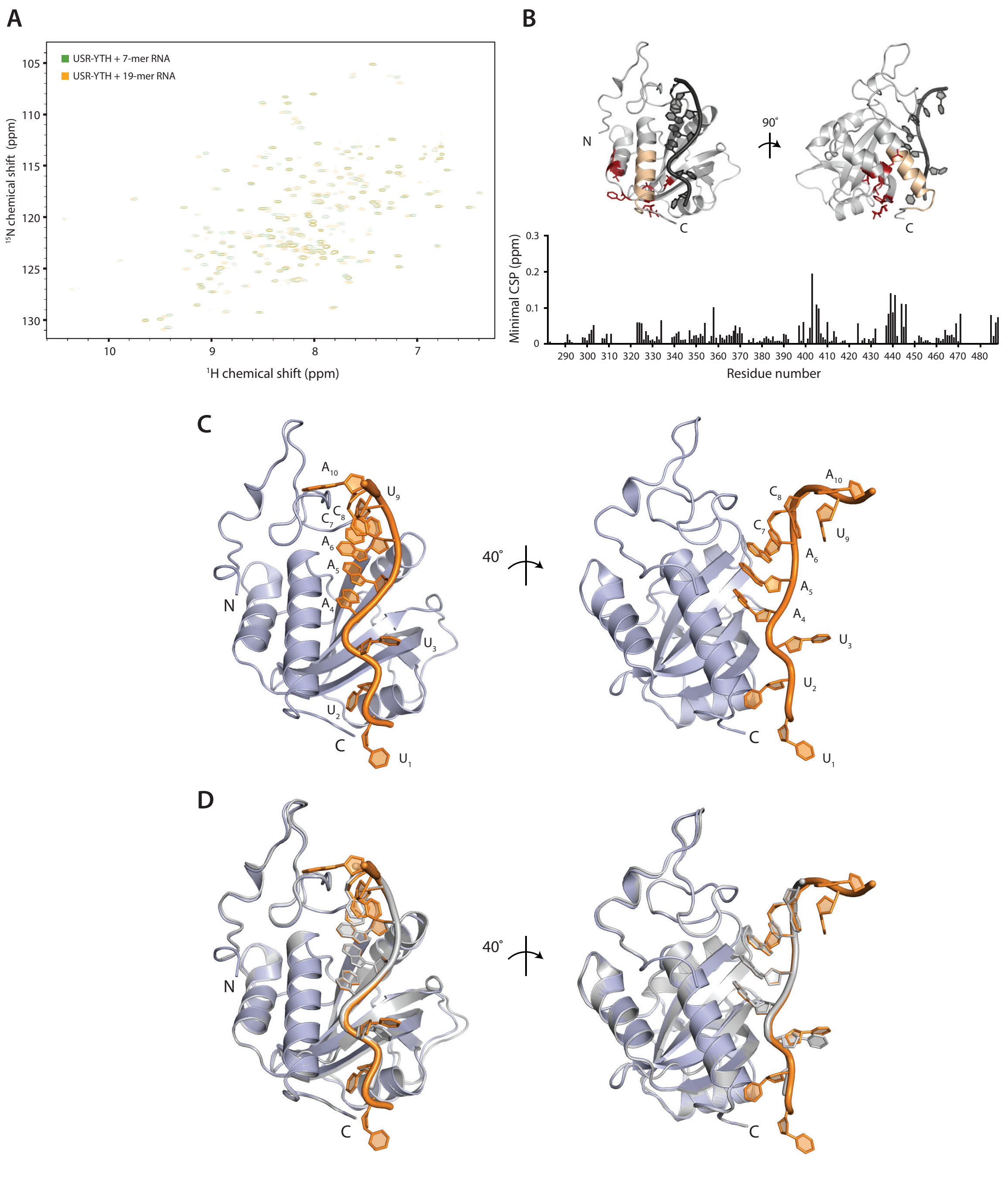

Figure S5. The low-complexity region is unaltered in the presence of longer RNAs. (A–B) 2D-NMR analysis of a USR-YTH construct bound to a 7-mer DSR sequence or a 19-mer DSR containing RNA. (A) Overlay of ^{1}H-^{15}N BEST-TROSY spectra. (B) Nearest neighbor chemical shift perturbation (CSP) maps (bottom). Regions showing large chemical shift differences (above 0.1 ppm; red) or line broadening (yellow) are mapped onto the crystal structure of the Mmi1 YTH domain (top). (C) Cartoon representation of co-crystal structure of Mmi1 residues 301–488 with an 11-mer RNA (CUUUAAACCUA). Rotated view shows that there are no further contacts outside the UNAAAC motif. (D) Structural alignment of the co-crystal structures of Mmi1 residues 301–488 bound to 7-mer and 11-mer RNAs (colored in grey and blue respectively).

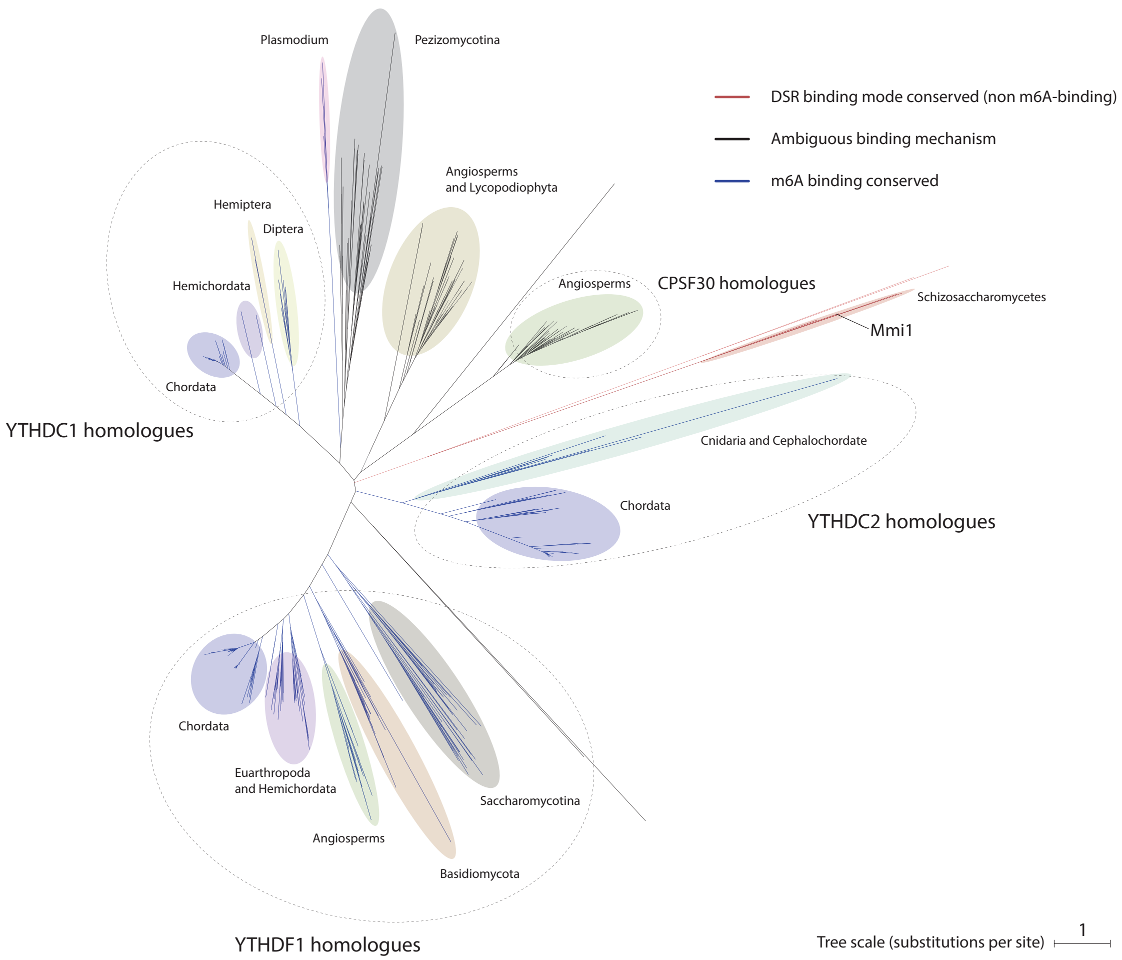

Figure S6. Evolutionary relationships of YTH domain-containing proteins. Unrooted phylogram of a non-exhaustive list of 1,097 YTH domain-containing proteins shows that Mmi1 occupies a distinct outgroup amongst otherwise structurally conserved domains in eukaryotes. Phylograms were constructed using a *MUSCLE* (2) sequence alignment and *FastTree* (3), then annotated both manually and using the *iTOL* server (4). Protein members of branches were inspected for conservation of m6A-binding (see Fig. S7). Three major lineages of 'canonical' m6A-binding proteins were identified as expected (YTHDF1, YTHDC1 and YTHDC2 homologues). The phylogram for members of these m6A-binding clades is colored blue. YTH domains with DSR binding modes like Mmi1 (as indicated by sequence analysis) are in red. Different taxonomies are shaded. Where m6A-binding residues were not conserved, organisms were examined for the conservation of the METTL3 methyltransferase complex (not found in *Schizosaccharomyces*). In Angiosperms, some species have the conserved methylation machinery, m6A-reading YTH domains, and YTH domains in CPSF30-like proteins, as well as divergent YTH domains. *Pezizomycotina* yeasts do not have a conserved methylation machinery and based on sequence analysis (Fig. S7) may also have a divergent RNA binding mechanism.

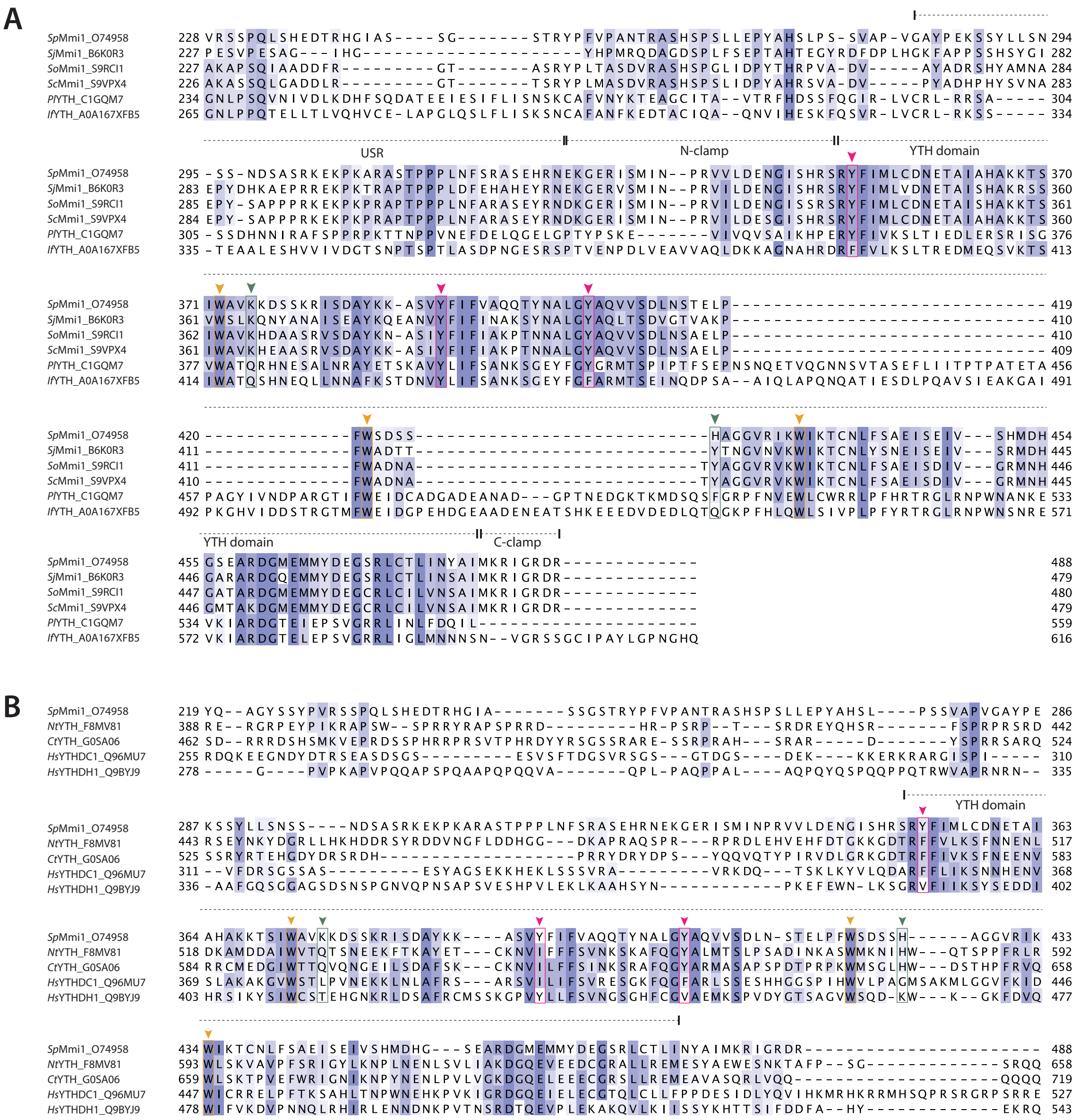

Figure S7. YTH domain sequence alignments. (A) Sequence alignments of *Schizosaccharomycetes* (*Sp*, *Schizosaccharomyces pombe*; *Sj, S. japonicus*; *So*, *S. octosporus*; *Sc*, *S. cryophilus*) show that parts of the USR are highly conserved in this genus. Alignments were performed in *Jalview* implementing *Clustal omega* (5, 6**)**. Two other YTH domains that are in the outgroup in the phylogenetic tree (*Pl*: *Paracoccidioides lutzii* and *If*: *Isaria fumosorosea*) (Fig. S6) do not show high conservation to Mmi1 upstream of the YTH domain. The core YTH fold tryptophan (W) residues are highlighted in yellow. These define the m6A-binding pocket but are also important for the YTH fold and are universally conserved in the YTH-domain superfamily. The tyrosine (Y) residues highlighted (magenta) form the Mmi1 DSR-binding platform on the conserved YTH fold. These residues are conserved in *P. lutzii* but not in *I. fumosorosea*. Residues highlighted in green are key residues in the m6A binding pocket that have diverged in Mmi1. K375 in *S. pombe* blocks access to the pocket and this is asparagine (Q) in *P. lutzii* and *I. fumosorosea* which could block substrate binding in the same manner. Similarly, a phenylalanine (F) in *P. lutzii*

(at position H426 in Mmi1) could also disrupt RNA-binding. (B) *Pezizomycotina* yeasts examined in this study possess YTH domains that may have a divergent RNA binding mechanisms, different from both m6A and DSR binding. Shown are sequence alignments of Mmi1 with, *Neurospora tetrasperma* (*Nt*) and *Chaetomium thermophilum* (*Ct*), and the m6A-binding *Homo sapiens* (*Hs*) YTHDC1 and YTHDF1. Key residues are labelled as described in (A).

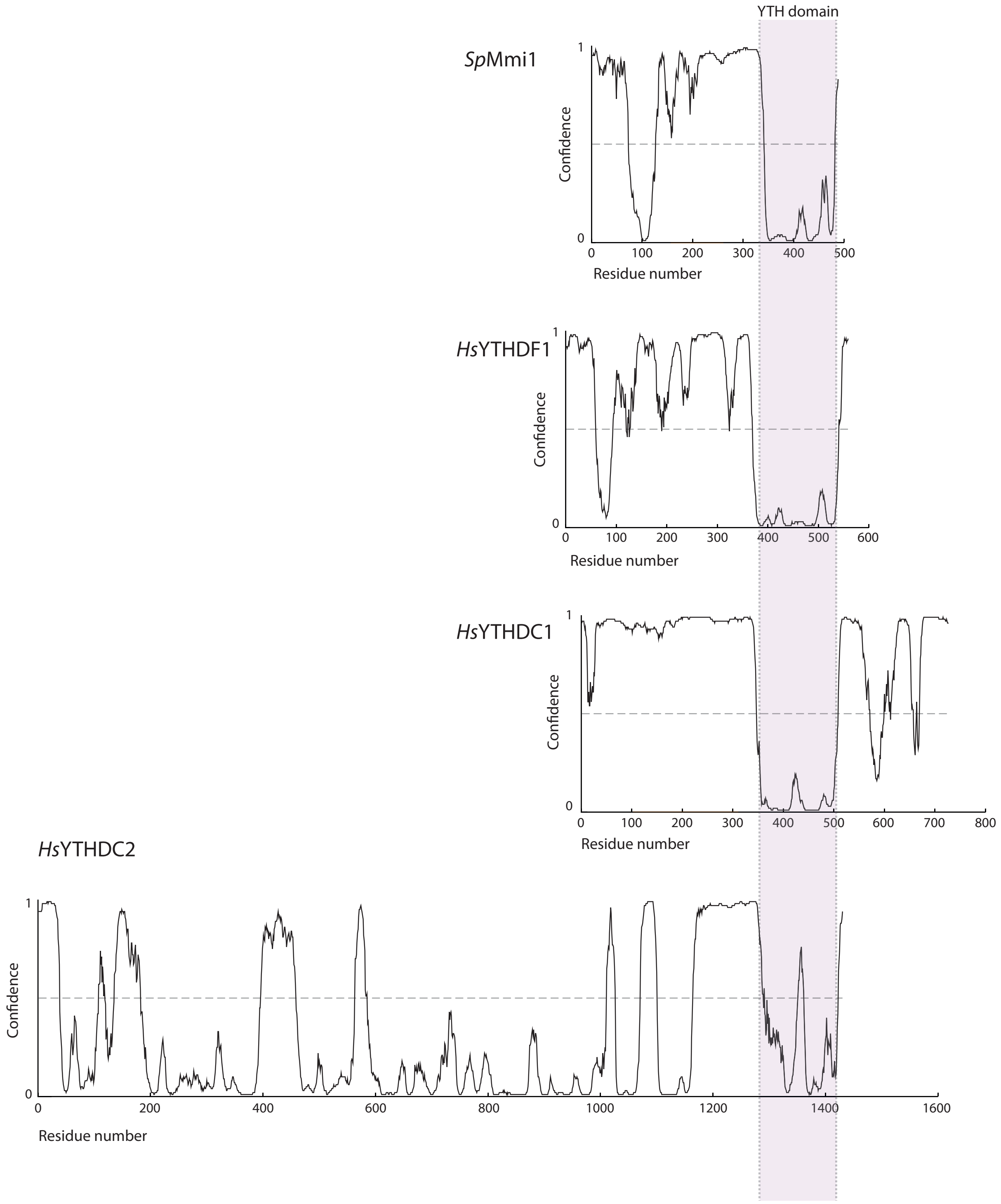

Figure S8. Most YTH domains have a proximal disordered region. Profiles of predicted disorder per residue are shown for *S. pombe* Mmi1, and human YTHDF1, YTHDC1 and YTHDC2. These represent distinct clades of YTH domain-containing proteins (Fig. S6). The profiles were created using *DISOPRED3* for the entire uniprot entry (7). Regions with confidence scores above 0.5 (horizontal grey dashed line) can be considered disordered. Plots are aligned by the location of the YTH domain (shaded purple).

Table S1 – Data collection and refinement statistics for USR-YTH Mmi1 crystallography

**Data Collection**	**Mmi1(327–488)** (PDB 6FPP)	**Mmi1(301–488) + 7mer RNA (PDB 6FPQ)**	**Mmi1(301–488) + 11mer RNA (PDB 6FPX)**
Wavelength (Å)	0.9763[1]	0.9677[2]	0.9795[3]
Space group	C 2	$P\ 6_5$	$P\ 3_2$
Unit cell parameters			
a, b, c (Å)	122.04, 58.30, 53.77	77.41, 77.41, 66.21	105.33, 105.33, 66.71
α, β, γ (°)	90.0, 114.7, 90.0	90.0, 90.0, 120.0	90.0, 90.0, 120.0
Resolution range (Å)	51.6–1.93 (1.98–1.93)*	47.11–1.42 (1.47–1.42)	91.22–1.97 (2.02–1.97)
Unique reflections	25,919 (1,906)	42,567 (4,135)	58,703 (4,049)
$\langle I/\sigma(I)\rangle$	7.7 (1.7)	13.6 (1.6)	8.5 (1.6)
R_{merge}	0.078 (0.459)	0.084 (1.159)	0.076 (0.588)
$CC_{1/2}$	0.996 (0.790)	0.999 (0.548)	0.994 (0.869)
Completeness (%)	99.9 (99.6)	99.9 (99.6)	99.8 (97.0)
Multiplicity	3.2 (3.1)	7.7 (7.0)	5.1 (4.7)
**Refinement**	(residues 331–485)	(residues 315–488)	(residues 312–488)
Resolution (Å)	51.6–1.93 (2.00–1.93)	47.11–1.42 (1.45–1.42)	45.61–1.97 (2.02–1.97)
Reflections used	25,882 (2,840)	42,527 (2,805)	58,635 (4,101)
R_{work}/R_{free} (%)	19.0 / 23.8	14.3/16.8	20.0/23.5
Ramachandran favored region (%)	97.2	98.9	98.0
Ramachandran outliers (%)	0.0	0.0	0.0
No. of non-hydrogen atoms			
Protein	2,557	1,518	4,193
Water	215	229	220
Ligands (incl. RNA)	25	159	600
Average B-factors			
Macromolecules	25.4	18.5	55.1
Water	34.7	33.3	49.2
Ligands	48.0	22.1	79.8
r.m.s. deviations			
Bond lengths (Å)	0.003	0.017	0.015
Bond angles (°)	0.56	1.59	1.42

[1]DLS i03 beamline
[2]ESRF ID30-A3 beamline
[3]DLS i02 beamline
* Values in parentheses refer to the highest-resolution shell

Supplementary references

1. Wilkins, D. K., Grimshaw, S. B., Receveur, V., Dobson, C. M., Jones, J. A., and Smith, L. J. (1999) Hydrodynamic radii of native and denatured proteins measured by pulse field gradient NMR techniques. *Biochemistry*. **38**, 16424–16431
2. Edgar, R. C. (2004) MUSCLE: Multiple sequence alignment with high accuracy and high throughput. *Nucleic Acids Research*. **32**, 1792–1797
3. Price, M. N., Dehal, P. S., and Arkin, A. P. (2009) FastTree: computing large minimum evolution trees with profiles instead of a distance matrix. *Mol. Biol. Evol.* **26**, 1641–1650
4. Letunic, I., and Bork, P. (2016) Interactive tree of life (iTOL) v3: an online tool for the display and annotation of phylogenetic and other trees. *Nucleic Acids Research*. **44**, W242–5
5. Waterhouse, A. M., Procter, J. B., Martin, D. M. A., Clamp, M., and Barton, G. J. (2009) Jalview Version 2--a multiple sequence alignment editor and analysis workbench. *Bioinformatics*. **25**, 1189–1191
6. Sievers, F., Wilm, A., Dineen, D., Gibson, T. J., Karplus, K., Li, W., Lopez, R., McWilliam, H., Remmert, M., Söding, J., Thompson, J. D., and Higgins, D. G. (2011) Fast, scalable generation of high-quality protein multiple sequence alignments using Clustal Omega. *Molecular Systems Biology*. **7**, 539–539
7. Jones, D. T., and Cozzetto, D. (2015) DISOPRED3: Precise disordered region predictions with annotated protein-binding activity. *Bioinformatics*. **31**, 857–863
